# Barriers to and facilitators of rehabilitation according to socio-economic status, after acute respiratory distress syndrome due to COVID-19: A qualitative study in the RECOVIDS cohort

**DOI:** 10.1371/journal.pone.0316318

**Published:** 2025-02-28

**Authors:** Mathilde Kléber, Nicolas Meunier-Beillard, Isabelle Fournel, Eléa Ksiazek, Marine Jacquier, Fiona Ecarnot, Jean-Philippe Rigaud, Pierre-Louis Declerq, Jean-Pierre Quenot, Marie Labruyère

**Affiliations:** 1 Service de Médecine Intensive-Réanimation, CHU Dijon Bourgogne, Dijon, France; 2 INSERM, CIC 1432, Module Épidémiologie Clinique, Université de Bourgogne-Franche Comté, Dijon, France; 3 DRCI, USMR, CHU Dijon Bourgogne, Dijon, France; 4 Equipe Lipness, Centre de Recherche INSERM UMR1231 et LabEx LipSTIC, Université de Bourgogne-Franche Comté, Dijon, France; 5 SINERGIES, University of Franche-Comté, Besançon, France; 6 Department of Cardiology, University Hospital Besancon, Besançon, France; 7 Department of Intensive Care, Centre Hospitalier de Dieppe, Dieppe, France; 8 Espace de Réflexion Éthique de Normandie, University Hospital Caen, Caen, France; 9 Espace de Réflexion Éthique Bourgogne Franche-Comté (EREBFC), Dijon, France; CHU Nantes, FRANCE

## Abstract

**Background:**

The COVID-19 pandemic may have compounded social disparities in access to healthcare, with possible deleterious consequences on the functional prognosis of patients after a stay in the intensive care unit (ICU). In the previous RECOVIDS study, we reported that despite comparable pulmonary sequelae and similar access to rehabilitation, socio-economically “vulnerable” patients had lower quality of life at 6 months after an ICU stay. We aimed to describe the barriers to, and facilitators of participation in rehabilitation, among patients from the RECOVIDS study, regardless of their socio-economic situation.

**Methods:**

Qualitative study using semi-structured interviews with adult patients admitted to ICU for PCR-proven SARS-CoV-2 infection, and who had acute respiratory distress syndrome (ARDS) or had received high flow nasal oxygen. In addition, patients had to have been living at home for the month prior to the interview and had to be proficient in French. Eligible patients were randomly selected, aiming to select the same number of socially deprived and non-socially-deprived patients. Interviews were transcribed for thematic analysis.

**Results:**

In total, 31 interviews were performed from 10/2021 to 01/2022; 16 with socially deprived, and 15 with non-deprived participants. Average age was 65.2 (±11.6) years. Four themes emerged from the analysis of the interviews, namely: (1) the impact of the patient’s professional and socio-economic situation; (2) the feeling that age and socio-economic situation influence access to rehabilitation; (3) a perception that the healthcare system was saturated, and that inequalities exist in access to rehabilitation resources; (4) perception of previous own health and expectations of post-resuscitation health status.

**Conclusion:**

A precarious socio-economic situation has a substantial impact on access to rehabilitation after ICU admission for ARDS caused by COVID-19. It represents a barrier to rehabilitation through the combined action of various social determinants that deserve to be detected early, in order to take appropriate action to ensure that the most socially vulnerable individuals can benefit from access to rehabilitation.

## Background

The SARS-CoV-2 virus can cause severe hypoxic pneumonia and acute respiratory distress syndrome (ARDS), and many of these patients will require admission to the intensive care unit (ICU). COVID-19 related ARDS was life-threatening in the early stages of the epidemic, although the prognosis progressively improved over the course of successive waves of COVID [1,2]. The clinical consequences of a stay in the ICU due to COVID do not appear to differ substantially from those of ICU stays due to ARDS of other etiologies [3–5]. Conversely, physical symptoms (notably muscle weakness) [6], psychological symptoms (anxiety, depression, post-traumatic stress disorder) [7], and cognitive impairment (memory loss, loss of verbal fluency, attention disorders, impaired executive function) [8], collectively known as post-intensive care syndrome (PICS) [9], all seem to be more severe and of longer duration after COVID-19 [10,11].

In parallel, the COVID-19 pandemic took a particularly heavy toll on socially disadvantaged populations [12–14]. This likely compounded existing social disparities in terms of healthcare access [15], with deleterious effects on the functional prognosis of patients after a stay in the ICU [16]. Indeed, at the individual level, the economic consequences of a stay in the ICU arising from loss of earnings, delayed return to work, or even job loss, can ultimately lead to situations of social isolation [17]. Similarly, even outside the pandemic context, there may be inequalities in access to specialized rehabilitation linked to the patients’ socio-economic status, and this may also affect medium and long-term prognosis [16]. It has been shown that intensive rehabilitation, initiated early (i.e. while the patient is still in the ICU), is beneficial after ARDS [18–24]. Surprisingly, this benefit has not been observed in terms of long-term mortality after rehabilitation implemented at discharge from the ICU [25].

In a study performed during the pandemic, Declercq et al reported in the RECOVIDS study that despite comparable pulmonary sequelae and similar access to rehabilitation, patients considered to be socio-economically “vulnerable” had lower quality of life at 6 months after an ICU stay [26,27]. This suggests that beyond purely medical and factual considerations, there are other, socially determined factors that explain why socially disadvantaged individuals fare less well at 6 months after ICU admission for COVID-related ARDS. We therefore sought to complement the quantitative findings of the RECOVIDS with a qualitative enquiry. The aim was to describe the barriers to, and facilitators of participation in rehabilitation, among patients from the RECOVIDS study, regardless of their socio-economic situation.

## Methods

This qualitative study was performed as part of the larger RECOVIDS study, whose methods and results have previously been reported elsewhere [26,27].

The RECOVIDS study was approved by the ethics committee “Comité de Protection des Personnes Sud Méditerranée II” on 10/07/2020 under the number 2020-A02014-35. In line with French legislation, participants provided consent to be contacted for a semi-structured, qualitative interview to explore their responses in greater detail. During the interviews for the present study, participants were informed about the study, and all provided written informed consent to participation, and to the use of illustrative quotes for the purposes of publication (after anonymization and translation).

The inclusion criteria for the main RECOVIDS study [26] were: adult patients admitted to ICU for PCR-proven SARS-CoV-2 infection, in any of the 30 participating centres. To be included, patients had to have undergone chest computed tomography (CT) scan at the initial phase of management; have ARDS diagnosed according to the Berlin 2012 definition [28] or have received high flow nasal oxygen with a flow of at least 50L/minute with FiO_2_ > 50% and a PaO_2_/FiO_2_ ratio ≤ 200. The main exclusion criteria included a walking perimeter < 50 m or WHO performance status 3 or 4; chronic respiratory insufficiency; patients not affiliated to the national health insurance system; and patients under legal protection.

**Fig 1:**
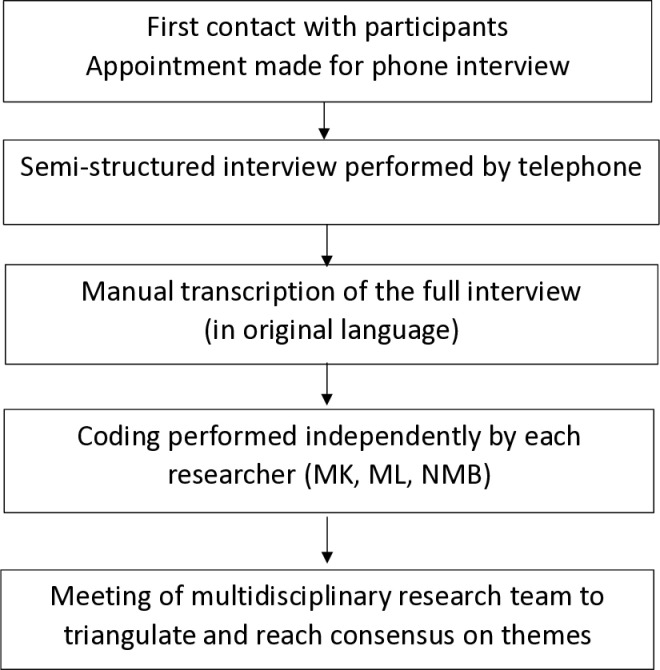
Illustration of the study procedures for data collection and analysis.

For the present qualitative study, additional inclusion criteria were as follows: Patients had to have been living at home for the month prior to the interview; they had to be proficient enough in French to perform the interview, and be free of any limitations on understanding or expression that would render the interview impossible. Eligible patients were randomly selected to be invited to participate; with the same number of participants selected from among socially deprived and non-socially-deprived patients, as assessed by the EPICES score [29–31]. The EPICES score is a measure of social and material deprivation. It assesses a total of 11 items covering social conditions, leisure activities and family/social support. Individuals with an EPICES score ≥ 30.17 (the lower boundary of the fourth quintile in the validation study) were considered to be socially deprived. To ensure maximum variation in the study sample, participants were randomly selected according to their level of social deprivation, as determined by the EPICES score. We also balanced the number of participants selected from the first and second pandemic waves (from January 2020 to July 2020 and from November 2020 to May 2021 respectively). The study procedures are summarized in Fig 1.

Next, the randomly sampled patients were contacted by 3 researchers from the RECOVIDS Qualitative team (MK, ML, NMB) to organize an individual, semi-structured interview at a time convenient for the interviewee. Interviews took place on average 20 months after discharge from the ICU.

The members of our multidisciplinary research group (bringing together specialists in public health, intensive care, respiratory medicine and sociology) held two meetings before the start of the study to review the current state of knowledge in the literature regarding rehabilitation after COVID-19 infection. Based on our review of the literature and expert knowledge, we developed the interview guide, which was reviewed and approved by the whole team. The interview guide focused on 4 main topics: (1) Barriers to rehabilitation and to recovering a quality of life that is perceived as satisfactory; (2) Facilitators of rehabilitation that is deemed to be efficacious by the patients; (3) availing of the resources offered by the national healthcare system; (4) the patient’s perception of their own physical and/or mental health. As the pandemic was still ongoing during the study period, all interviews were performed by telephone. Interviews were performed until data saturation was reached (i.e., the point beyond which further interviews yielded no new information). Interviews were recorded and fully transcribed (in French) for later analysis. Data were encoded to guarantee the anonymity of the participants.

Once all interviews had been completed and data saturation was reached, the research team held two more meetings to review the interview transcripts, triangulate coding and themes, and reach a consensus on the final thematic analysis. The transcripts of all interviews were analyzed using thematic analysis, as previously described [32–34]. In thematic analysis, the verbatim are first coded, then codes are regrouped into major and minor themes. Major themes are significant points that are mentioned spontaneously and well developed by all participants in a cross-sectional manner. Minor themes are secondary points that are less well developed, and spontaneously mentioned by some, but not all participants. The first level of analysis was first performed independently by each researcher (MK, ML, NMB) on their own interviews, then meetings were held to harmonize the categories and reach consensus on the themes to be retained. Results were discussed until consensus on interpretation was reached. Translation was performed after the results were finalized.

## Results

A total of 42 patients were contacted, of whom 31 (73.8%) agreed to participate in the interview (20 men, 11 women). Those who did not participate either refused to be interviewed, or failed to answer the call after scheduling an interview. Thus, a total of 31 interviews were performed between October 2021 and January 2022; 16 with socially deprived, and 15 with non-deprived participants. At that point, saturation was reached in the data, and thus, no further interviews were performed. Eighteen patients had been hospitalized during the first COVID-19 wave, and 13 in the second wave. The sex ratio was 4 women to 5 men for patients from the first wave, and 2:3 for the second wave. Median age was 58 years (interquartile range 17.5). The average duration of the interviews was 50.3 ± 7.1 minutes. The characteristics of the study participants are detailed in Table 1.

**Table 1:** Characteristics of the study population.

Interview number	Sex	Age	Social Deprivation	CSP	Highest educational qualification	Covid wave
1	Male	69	No	Business manager	High-school diploma	1
2	Female	75	No	Retired	Middle-school certificate	1
3	Male	80	No	Retired	Bachelor’s degree	1
4	Male	55	No	Manager/higher-level professional	Bachelor’s degree	1
5	Female	77	No	Retired	Bachelor’s degree	1
6	Male	64	No	Retired	Bachelor’s degree	1
7	Male	73	No	Retired	Vocational training certificate	1
8	Female	58	No	Manager/higher-level professional	Bachelor’s degree	1
9	Female	56	No	Manager/higher-level professional	Bachelor’s degree	1
10	Male	65	Yes	Blue-collar worker	Vocational training certificate	1
11	Male	70	Yes	Retired farmer	Vocational training certificate	1
12	Male	49	Yes	Blue-collar worker	High-school diploma	1
13	Female	47	Yes	Office employee	Vocational training certificate	1
14	Female	51	Yes	Blue-collar worker	High-school diploma	1
15	Male	57	Yes	Blue-collar worker	Vocational training certificate	1
16	Male	67	Yes	Retired	Primary school	1
17	Male	56	Yes	Intermediary profession	High-school diploma	1
18	Female	51	Yes	Blue-collar worker	High-school diploma	1
19	Male	63	Yes	Unemployed	Vocational training certificate	2
20	Female	48	Yes	Office employee	High-school diploma	2
21	Female	71	Yes	Retired farmer	Vocational training certificate	2
22	Male	50	No	Trader	Bachelor’s degree	2
23	Male	59	No	Manager/higher-level professional	Vocational training certificate	2
24	Male	61	No	Business manager	Bachelor’s degree	2
25	Female	53	No	Manager/higher-level professional	Bachelor’s degree	2
26	Male	49	No	Craftsman	High-school diploma	2
27	Female	51	No	Manager/higher-level professional	High-school diploma	2
28	Male	56	Yes	Farmer	Vocational training certificate	2
29	Male	58	Yes	Intermediary profession	Vocational training certificate	2
30	Male	74	Yes	Retired blue-collar worker	No qualification	2
31	Male	78	Yes	Retired blue-collar worker	Vocational training certificate	2

Four themes emerged from the analysis of the interviews, namely: (1) the impact of the patient’s professional and socio-economic situation; (2) the feeling that age and socio-economic situation influence access to rehabilitation; (3) a perception that the healthcare system was saturated, and that inequalities exist in access to rehabilitation resources; (4) perception of previous own health and expectations regarding post-resuscitation health status.

**Fig 2:**
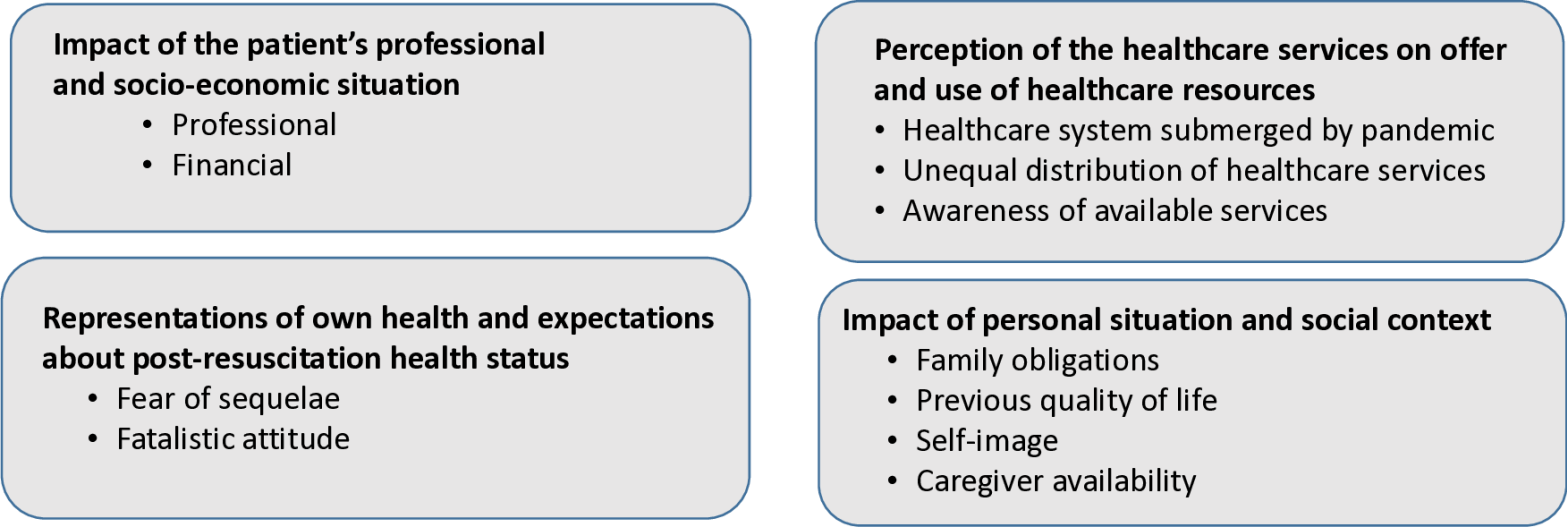
Key findings from the study in each theme.

Illustrative quotes for each theme are given in Table 2, and the main themes are summarized in Fig 2.

**Table 2:** Illustrative quotes for each theme.

ID	Quote
**Theme 1: Impact of professional and socio-economic situation**	
N°12	*“When you have two children to feed, you can’t afford to be going on sick leave every time you get a pain or every time you feel stressed”*
N°14	*“I immediately asked the doctors to let me out [of hospital] so that I could go back to work”*
N°15	“*If I had the time to do some training, I think that’s what I would do”*
N°13	*“I have enough problems as it is with my family and my job without trying to think about that [i.e., training/reconversion] as well”*
N°1	*“I couldn’t abandon everyone. There were things that I hadn’t sorted out in my personal affairs and in my business.”*
N°4	“*It was also a question of dignity for me. How do you feel when you look in the mirror if you’re not working?”*
N°24	*“I’m focused on the material aspects, because I want my business to flourish and I want to be able to pay my employees at the end of the month”*
N°4	*“I still work because I love my job and indeed, that’s what pushed me to get better”*
N°2	*“It’d break my heart to give up my job, because as well as being a job all my life, it’s also my passion”*
N°26	*“For sure, when you have a good situation in life, it gives you a goal, you know what you’re fighting for, to try and get well again and be capable again”*
N°31	*“What can I do? I’d already stopped doing anything interesting before, so a little more or a little less now, it doesn’t make much difference”*
N°11	“*I already get social welfare assistance to pay my rent, they can hardly pay for a bike for me as well, just for my leisure”*
N°17	*“The least you can do it pay some out-of-pocket expenses for your health, for example, the home bike, I saw some that were quite suitable and not at all expensive”*
N°29	*“It’s not so much that you can’t pay for things like a bike or for sports activities, it’s just that you’ve nothing left to live on, so you have to go back to work for that”*
N°21	*“When you have to be careful already so that you have enough to eat, you definitely can’t be thinking about stuff like sport, which is important, I know, but not as vital”*
**Theme 2: Impact of personal and social context on access to rehabilitation**	
N°9	*“It’s easier when you have other, outside motivations, other than just your dignity”*
N°2	*“I couldn’t bear the idea of being a burden for my family”*
N°3	*“You go right back to zero, you can’t do a single thing, not even go to the toilet, without help, it’s awful”*
N°5	*“I really tried to work hard and recover, to show my granddaughters that there’s always hope”*
N°7	*“My plan of action was to get back to a good level of physical health, so that I could put my affairs in order, then take it easy, retire, and spend quality time with my family”*
N°18	*“I had a small amount of welfare assistance from one of my interim contracts, but it was only 300 Euro a month and you can’t take care of a family with 2 kids on that. It’s not even enough for one person on their own.”*
N°28	*“I might be just a farmer, but I have my dignity, I was doing even less than my animals… what would I be like if I stayed like that?”*
N°30	*“My children still need me, even though they’re grown up. I can’t leave them, it wasn’t an option, I just had to get better”*
**Theme 3: Perception of healthcare system**	
N°20	*“With all those patients getting out of ICU all over the region, it was much more difficult, and they [the caregivers] didn’t have as much time”*
N°16	*“Communication isn’t always optimal between the different staff members, and the goals they should be reaching aren’t always clear, with the result that they don’t see what they’re working for”*
N°31	*“You have to at least have a certain level of autonomy but I’m still not able to get around much”*
N°22	*“The home bike? I don’t see how I could even get up on it, in the state I’m in at the moment”*
N°3	*“I got the feeling that, for people like me who are really very impacted, the rehabilitation programme wasn’t optimal”*
N°28	*“The pain was stopping me from working more – I mean more intensely and for longer”*
N°16	*“I wasn’t really able to do the exercises, and at the beginning, it was painful”*
N°30	*“They didn’t have that in the rehabilitation centre where I was, you can clearly see that it’s only the hospital’s “poor cousin””.*
N°29	*“My wife doesn’t drive, I can’t drive myself any more, my children aren’t around every day, and I live in a small village that’s quite far from the rehabilitation centre”*
N°25	*“There was quite a long time between when I got out of the ICU, and when I started rehabilitation”*
N°3	*“All that time when I wasn’t moving enough and I was doing nothing, well that put me behind, and then you can never really catch up”*
N°11	*“They asked me if I wanted to do rehab, but I just wanted to go home. I’d already been in the hospital too long, I just wanted to go home”*
N°27	*“The famous hospital room, with the bed in the middle, the standard-issue night table, the beige-white walls…. after a while it’d drive you crazy”*
N°21	*“They set up assistance before I got out, with home help for the housework, a meal delivery service, and a medical bed downstairs. Otherwise I wouldn’t have been able to go home”*
N°26	*“There was a huge gap compared to when I was in hospital for the rehabilitation and I was seeing the physiotherapist 4 times a day, and the ergotherapist, and the psychomotor therapist and all the rest”*
N°23	*“When you go home and you only have 3 physiotherapy sessions a week and that’s all, well it’s true that there’s a huge difference compared to being in rehabilitation”*
N°27	*“I was just pleased to get home. That’s where I did the most rehabilitation”*
N°18	*“When you’re in the hospital, you have physiotherapy, special equipment to work with, they give you your food, they do the cleaning, you have nothing to do. You don’t have to move about because they do everything for you, but when you go home, well…. You have to go and wash yourself, and do the housework and take care of things”*
N°9	*“In France, it’s seen as a weakness if you have to go to a psychologist. Drugging yourself with anti-anxiety meds, that’s accepted by society, but going to see a psychologist is a problem. The results of rehabilitation could be greatly improved with the use of psychology”*
N°24	*“The psychology did me a lot of good because it enabled me to focus on something other than the physical difficulties I had after the stay in the ICU”*
N°27	*“If I hadn’t had psychological help, I think I would have been absorbed by my anxiety and by what happened to me, I would’ve been ruminating about it”*
N°15	*“Once I got out of rehabilitation, I had to manage on my own”*
N°28	*“I don’t have a GP, so if I go to the doctor, it’s because there’s a problem – it’s not to be asking him for physiotherapy or massage sessions”*
**Theme 4: Perception of own health**	
N°22	*“Not being able to move or staying in bed like a vegetable has always been my biggest fear”*
N°6	*“I would’ve preferred to die rather than be so diminished, I would even go so far as to say incapable, because I wasn’t diminished in the sense of my activities being limited – I actually could do nothing at all”*
N°3	*“You can’t work miracles either. My age is what it is, it’s normal for things to be more difficult after more than a month in an artificial coma”*
N°28	*“You know, us farmers, we don’t last too long”*
N°4	*“I’m still alive so the goal has been reached”*
N°17	*“If I had died, I wouldn’t be able to do anything at all, so even if I get tired now and I have to rest a bit more, it doesn’t really matter”*
N°10	*“When you go in there [ICU], it means you’re hovering between life and death, so today I’m on the life side, and that’s the right side to be on, that means things worked out”.*
N°3	*“For me, being in good health means not being impaired, and being able to do whatever you want without having to say yourself, I’m going to be out of breath, or it’s going to be painful. For the time being, that’s not the case for me yet”*

### Impact of the patient’s professional and socio-economic situation

#### Professional situation.

Patients who were classed as socially deprived mostly had low-level jobs (low level of qualifications, interim jobs, absence of social security benefits). For these patients, this was actually a strong motivation to undertake intensive rehabilitation, but because of their social situation, could also mean that they could only participate for shorter periods.

Also, socially deprived patients reported obstacles to changing careers or jobs, or to adapting their current position to their illness. Some even felt this would totally impossible.

Among the group of patients who were not socially deprived, occupying positions of responsibility was a motivating factor for compliance with well-implemented rehabilitation, especially among the participants with a high level of education. Similarly, it was important for them to recover good physical and mental capacity, with a view to a rapid return to work.

In this group, a satisfying professional situation was also a source of motivation, as the patients underlined the importance of wanting to recover their previous level of autonomy and quality of life, so that they could go back to work.

#### Financial situation.

A corollary of a precarious professional situation is that many socially deprived participants also had financial difficulties, and this was also seen among retired persons. Insufficient financial resources is a key explanation for the lack of personal motivation to engage in health-promoting behaviours, especially things like renting or buying rehabilitation equipment with a view to pursuing rehabilitation at home.

Conversely, non-socially deprived participants feel that it is important to be personally implicated in the rehabilitation process.

### Impact of personal situation and social context

The patients’ quality of life and personal activity prior to ICU admission was very frequently mentioned by non-socially deprived participants, as a source of personal motivation to recover.

All patients, regardless of their socio-economic group, underlined the importance of their self-image, especially the loss of autonomy and dependency on the healthcare providers at the start of their hospitalization.

The presence of an informal caregiver seemed to act as a facilitator of participation in rehabilitation, because of the caregiver’s important role in the logistic organisation (e.g., transport to rehabilitation centre, purchasing equipment, assisting with housework etc). Among non-socially deprived participants, the presence of an informal caregiver in the entourage was an additional motivation to do their best to achieve full recovery, in order to be able to spend quality time with their family once again.

Among socially-deprived individuals, family obligations (e.g., single parents, or families with a lot of children; or still supporting grown-up children who are not financially independent) were a motivating factor to recover the previous level of capacity as quickly as possible, but were also a practical obstacle to long-term participation in rehabilitation.

### Perception of the healthcare services on offer and use of healthcare resources

The pandemic context led to an abrupt, and immense increase in the workload of caregiving teams in healthcare establishments everywhere, and this led to organisational difficulties in healthcare delivery. This was perceived by the patients.

Non-socially deprived participants also highlighted a mismatch between the availability of rehabilitation programmes, and their sequelae, notably neuro-muscular deficits or chronic pain.

Regardless of their socio-economic situation, all the participants mentioned the unequal distribution of healthcare services across the country (e.g., private practice healthcare professionals, available healthcare establishments). Conversely, geographical distance was mainly cited as a barrier by those who were socially deprived, especially those who had little or no access to transport.

For some patients, there was also a long time interval between their discharge from ICU, and their entry into rehabilitation, mostly linked to the presence of medical complications that required long and complex management, which relegated rehabilitation to lower priority.

A prolonged stay in hospital was also a factor associated with non-adherence to rehabilitation, and especially for socially disadvantaged patients, raised the question of outpatient or ambulatory management. Outpatient management could encourage better adherence, by removing the potential negative repercussions of hospitalization on the patient’s social, family and professional life, while also limiting the strain on hospital resources.

The return home was seen by all patients as a key turning point, where the healthcare on offer was often found lacking, especially if the patient had had a prolonged period of intense physical rehabilitation.

Housework and duties in the home, often unavoidable, are often the cause of premature discontinuation of medical follow-up and/or rehabilitation. Indeed, all the patients, regardless of their socio-economic situation, underlined the time and energy required to do basic housework, and tend to personal and family obligations.

The utility of mental health support was also underlined by all participants, regardless of their socio-economic situation:

Finally, there were reported differences in access to care between socially-deprived and non-socially-deprived patients. The more affluent patients knew the different types of care and services on offer (physiotherapy, osteopath, psychologists, ergotherapy…) and did not hesitate to ask for these services when in rehabilitation, whereas socially deprived patients had less knowledge of available services, and used them much less frequently after leaving hospital. This likely reflects a higher level of education and higher health literacy among the more affluent participants.

### Representations of own health and expectations about post-resuscitation health status

For all participants, the fear of motor and respiratory sequelae that might impact on their future autonomy was a strong motivating factor to initiate and pursue rehabilitation, with a view to recovering the quality of life they had had prior to becoming ill with ARDS.

Among older participants, regardless of their socio-economic situation, and among the socially deprived younger participants, there was a fatalistic attitude that could lead to a lack of motivation to undertake rehabilitation, since their disease was no longer life-threatening.

## Discussion

In this qualitative substudy based on the multicentre RECOVIDS project [26], we performed semi-structured interviews with patients who had been admitted to ICU for ARDS due to COVID-19. Analysis of the discourse of these patients reveals that there are numerous barriers to the implementation of rehabilitation after a stay in the ICU, and these are predominantly related to the patient’s professional status, age and socio-economic situation. Conversely, geographic proximity to a rehabilitation centre, early ambulatory management, a supportive entourage or a stable professional situation were all facilitators of initiation and pursuit of rehabilitation. Regardless of their socio-economic situation, all the patients interviewed were acutely aware of the saturated state of the healthcare system during the pandemic, which sometimes precluded timely (or any) access to rehabilitation centres or specialist consultations. Consequently, some patients perceived a shortfall between the offer of care, and their persisting sequelae (e.g., neuro-muscular disorders), or experienced a long delay between discharge from ICU and access to rehabilitation.

Social deprivation can result from the cumulated breakdown of social, professional and/or emotional support. In socio-economic terms, it implies the absence of security in one or several domains, notably employment. This in turn precludes the individual from fulfilling their professional, family and/or social obligations, and from enjoying their basic rights to the full, notably the right to health. Healthcare inequalities are the reflection of the link between health and social determinants, and differ widely from one country to another, in terms of access to care and health insurance coverage [35]. In addition to socio-economic situation, numerous social determinants and individual behaviours combine to make healthcare inequalities largely avoidable. In health terms, healthcare inequalities are truly a loss-of-opportunity for the most socially disadvantaged populations, as was previously observed during the COVID pandemic [12–14]. It is noteworthy that healthcare inequalities generally reveal the weaknesses of a healthcare system and a social gradient linked to social disadvantage, and they have an impact both upstream and downstream of a stay in the ICU [16]. Accordingly, low income and a low level of education have been identified as socio-economic factors that aggravate the severity of COVID-19, compounding socio-economic vulnerability, which in turn may be responsible for co-infection (e.g. tuberculosis), malnutrition or risk-taking behaviours (alcohol/tobacco consumption, addiction) [36]. It has also been reported that ICU admission may have negative financial consequences for 33% of patients at 6 months, and 28% at 12 months [37], and this can create additional socio-economic difficulties for patients. In France, available data regarding health inequalities come from ICU populations [38,39]. In the IVOIRE study [39], social deprivation, evaluated by the EPICES score, was observed in 48.5% of patients and was shown to influence the risk of nursing home entry within 3 to 6 months after a stay in the ICU. In the FROG-ICU study [38], the authors observed a significant decline in physical capacity, evaluated by the Medical Outcomes Study 36-item Short Form at 1 year among patients with a high level of social disadvantage, as assessed by the French deprivation index.

A previous review of the literature that included studies of the consequences of ICU admission also found socio-economic issues to be foremost for most patients [40]. The issues were manifest in a variety of ways, including changes in relations to others (family, friends, colleagues) with a tendency to become socially isolated [41]; the feeling of being a burden for the family [42]; the feeling of being limited in daily life by restrictions laid down by others, or even a feeling of uselessness [43]. Conversely, for patients in psychological distress, the presence of the family is of utmost importance [44], as is the presence of a supportive and assisting social environment [33], in the process of recovery. The utility of psychological follow-up, and the desire to return to the life they had before the illness, were both highlighted in our study, especially by the socially deprived group. Indeed, the capacity to fulfil family roles and participate in social activities has a strong influence on perceived quality of life [40]. By the same token, an inability to return to previous activities and/or resume their prior professional status is considered as a form of handicap that can cause considerable psychological distress [45].

Other authors [46,47] have suggested that there is a strong link between precarity and outcome among patients admitted to ICU, notably due to greater difficulty accessing care in geographically distant patients, a tendency to wait longer before seeking care [48], and a higher level of social deprivation according to race or ethnic origin [49]. The outcome of patients after ICU discharge is to a large extent influenced by the degree of disability they suffer, and how impaired they are for the activities of daily living [50]. In this regard, Ferrante et al reported that, compared to life prior to ICU admission, there was a marked increase in severe disability, which went from 26% before, to 51% after ICU, with significant functional decline at one year [51].

In our study, the social environment (family commitments, housework, job), financial situation and available family support were all important determinants of the patient’s commitment to rehabilitation after their ICU stay. Indeed, our interviews revealed that logistic and geographical barriers are less important than the more preponderant social aspect. Indeed, socially disadvantaged patients were out of touch with the healthcare system once they left the hospital or rehabilitation. In our study, this phenomenon was due to various factors including financial precariousness and the need to return to work to maintain their income, but also a lack of awareness of the care on offer within the healthcare system, such as outpatient care. Finally, some patients also had a fatalistic attitude regarding their health, that paralleled their social situation. Cohen and Wills previously underlined the negative impact that social disadvantage may have on an individual’s capacity to cope with a stressful event and mobilize available resources [52]. Job precarity can make some individuals more likely to lose their job or income when a major health event befalls them [53], and exposure to job precarity is associated with negative health outcomes that may compound health inequalities at population level [54]. A key challenge after a stay in the ICU, particularly at the time of the patient’s return to home, is to detect the components of PICS, which can be done using specific instruments [55,56] or during a dedicated post-ICU consultation. This can help to guide rehabilitation accordingly [20,22] and help the patient to recover an acceptable quality of life [57]. Implementation of multidisciplinary programmes for outpatient rehabilitation after ICU discharge have been shown to improve quality of life at one year, and enable an earlier return to work [58]. Similarly, the input of social workers can be particularly helpful for both patients and their caregivers [59,60]. In France, social workers have a key role in organizing the return home after hospitalization, and their scope of intervention is specifically focused on supporting precarious social situations, such as individuals exposed to job or accommodation precarity, loss of income or complex family situations.

Providing rehabilitation in the home can be an attractive option for those who require rehabilitation but who may experience difficulties in accessing rehabilitation services in a hospital-based or specialized environment. In a recent review of the literature, Velez et al describe the factors that influence the provision of home-based rehabilitation services [61]. The authors found that patients find home-based services to be more convenient and less disruptive of their everyday lives, while at the same time empowering them for self-management. However, home-based provision of rehabilitation poses challenges for privacy and confidentiality across the different stakeholders (patients, family, care providers….). Other factors were highlighted in the quest to expand home-based rehabilitation, including support from providers and family members, the need for good communication with healthcare providers, the requirements made of patients and their surroundings, whether the patient lives alone or not, and the availability of both providers and equipment [61,62]. Many of these same issues were highlighted by the respondents in our study. A final factor concerns the transition from hospital-based to home-based services, which may be difficult for some patients [61]. Indeed, as reported in our study, some patients may have the impression that they are less well cared for when services are provided in the home, because of a lack of human and material resources, giving patients the impression that they have been abandoned once they are discharged from hospital. All these points need to be taken into careful consideration when evaluating the feasibility of home-based rehabilitation services.

### Study limitations

This study has some limitations. As with all qualitative studies, there may be potential for social desirability bias, whereby patients will say what they think the interviewer wants to hear, or may be reluctant to say things that might be socially unacceptable. This bias was minimized by asking patients to describe concrete examples from their personal experience, and by assuring the patients that the interviewers were independent, not involved in the rehabilitation, and that all data would remain strictly confidential. Secondly, the interview guide may not have covered the full spectrum of possible issues that could arise in relation to the subject. Third, we chose to interview both patients who were socially deprived, and those were not, as categorized in our previous randomized, controlled trial. This choice may limit the generalizability of the results to the whole population of patients admitted to ICU. Finally, we only interviewed patients who had been home for at least one month and who were able to express themselves articulately. This may leave room for selection bias since the most severely affected patients were not included.

## Conclusion

A precarious socio-economic situation has a substantial impact on access to rehabilitation after ICU admission for ARDS caused by COVID-19. It represents a barrier to rehabilitation through the combined action of various social determinants that deserve to be detected early, in order to take appropriate action to ensure that the most socially vulnerable individuals can benefit from access to rehabilitation. We suggest that early detection of social vulnerability, e.g., at admission of patients, should be performed, using validated tools, which will necessarily need to be different, and validated, for each individual country. The evaluation of social vulnerability should take account of existing solutions for support (financial and other) within each country’s healthcare and social welfare system. This would maximise the support for each patient according to their needs, and optimise the conditions of their discharge, enabling them to return home with appropriate support, or to transition to affordable rehabilitation and convalescence centres. This could contribute to reducing health inequalities in the population, which are already known to be associated with social disadvantage.
